# Hospitalization Predictors in Acute Exacerbation of Chronic Obstructive Pulmonary Disease: A Post Hoc Study of a Multicentric Retrospective Analysis

**DOI:** 10.3390/jcm14082855

**Published:** 2025-04-21

**Authors:** Grgur Salai, Tatjana Tokić Vukan-Ćusa, Mirna Vergles, Sanda Škrinjarić Cincar, Jelena Ostojić, Matea Škoro, Žarko Vrbica, Emilija Lozo Vukovac, Neven Tudorić, Andrea Vukić Dugac

**Affiliations:** 1 Department of Pulmonology, Dubrava University Hospital, 10000 Zagreb, Croatia; salai.grgur@gmail.com (G.S.); tatjana.tvc@gmail.com (T.T.V.-Ć.); mirna_korica@yahoo.com (M.V.); 2 Faculty of Medicine, University of Osijek, 31000 Osijek, Croatia; sanda.skrinjaric.cincar@mefos.hr; 3 Clinic for Respiratory Diseases, University Hospital Centre Zagreb, 10000 Zagreb, Croatia; jelena.ostojic.zg@gmail.com (J.O.); skoro.matea@gmail.com (M.Š.); 4 Department of Internal Medicine, Dubrovnik General Hospital, 20000 Dubrovnik, Croatia; zvrbica@yahoo.com; 5 University Study Programme Nursing, University of Dubrovnik, 20000 Dubrovnik, Croatia; 6 Department of Pulmonology, University Hospital Split, 21000 Split, Croatia; emilija.lozo@gmail.com; 7 Pulmonary Outpatient Clinic, St. Catherine Specialty Hospital, 10000 Zagreb, Croatia; neven.tudoric@gmail.com; 8 School of Medicine, University of Zagreb, 10000 Zagreb, Croatia

**Keywords:** COPD, acute exacerbations of COPD, predictors, discharge record, post hoc analysis

## Abstract

**Background/Objectives**: Hospitalizations for acute exacerbations in patients with chronic obstructive pulmonary disease (AECOPD) are connected with considerable mortality and morbidity and have a great impact on healthcare costs. We aimed to identify potentially important characteristics that distinguish patients with AECOPD that required hospitalization between those that did not. **Methods**: We performed a post hoc study of a previously conducted observational study assessing the discharge records of AECOPD patients who were either hospitalized or discharged directly from the emergency department (ED). **Results**: A total of N = 593 AECOPD patients (37.4% female) were included: N = 225 (37.9%) were hospitalized, while 368 (62.1%) were discharged from the ED. Patients had a mean age of 71 (±9.14) years. Further, 64.7% had arterial hypertension, and 60.4% of hospitalized and 42.1% of discharged patients had cardiovascular disease (excluding hypertension) (*p* < 0.001). In addition, 24% of hospitalized patients suffered from diabetes mellitus (vs. 16.8% of those discharged, *p* = 0.042). Patients that were discharged had a higher median eosinophil count than hospitalized patients (*p* < 0.001). Among the 368 patients discharged from the ED, 23.6% visited the ED due to AECOPD, and 50.6% were hospitalized in the subsequent three months. Patients that had at least one AECOPD in the subsequent three months had significantly lower initial eosinophil counts than those that did not (*p* = 0.015). **Conclusions**: Hospitalized AECOPD patients had a higher prevalence of pre-existing cardiovascular disease, diabetes mellitus and lower blood eosinophil counts. Patients that had subsequently visited ED in the following three months initially had lower blood eosinophil counts than those that did not make subsequent visits.

## 1. Introduction

The acute exacerbation of chronic obstructive pulmonary disease (AECOPD), as defined by the Global Initiative for Chronic Obstructive Lung Disease (GOLD), is an event characterized by increased dyspnea and/or cough and sputum that worsens in less than 14 days. This event is often associated with local and systemic inflammation caused by various insults to the airways, such as infection, pollution, etc. [1].

AECOPDs are events associated with significant mortality, impaired health status and socioeconomic burden [2,3]. They lead to the loss of lung function, and previous AECOPDs are one of the major risk factors for future exacerbations [4,5,6,7,8]. Notably, following an AECOPD, patients are at an increased short- and long-term risk of (major) cardiovascular events [9,10].

Acute exacerbations are heterogenous in etiology. Several attempts have been made in order to identify the etiological phenotypes of AECOPD [11,12,13]. MacDonald et al. conducted a study, in which they identified 26 distinct etiological phenotypes and found multifactorial etiology in about 70% of patients [14].

“Frequent exacerbator” has been recognized as a distinct COPD phenotype, associated with poor clinical outcomes. Patients with this phenotype are more likely to have increased hospital admissions and are associated with increased mortality [15,16]. Uslu et al. found that frequent exacerbators tend to be older and have lower eosinophil counts in peripheral blood than other COPD patients [17].

The question of distinguishing between AECOPD patients requiring hospital management and those that may safely be discharged from the ED still remains largely unanswered, as AECOPD severity is usually graded post hoc, based on the decision of hospital management. The Rome Proposal was established using the Delphi method in order to classify AECOPD severity based on clinical signs and symptoms, biochemical markers and arterial blood gas analysis [18]. Real-world data, in which in-hospital mortality was observed according to the Rome Proposal stratification system, yielded promising results [19]. However, this system is not widely used (in Croatia), and the decision-making safety is yet to be demonstrated.

Despite the progress being made in the study of AECOPD, no clear guidelines for exacerbation phenotyping and subsequent management have been established. Additionally, no clear international recommendations to distinguish between patients that require hospital admission and those that do not are available. National and regional data regarding the characteristics of patients that are chronic obstructive pulmonary disease (COPD) “exacerbators” are lacking. Considering the specificities of the Croatian healthcare system, where basic care for COPD patients is the task of family practitioners, in our previous study, we analyzed emergency department (ED) and hospitalization discharge letters from the pre-COVID-19-pandemic era in order to observe to what extent the recommendations of discharge summaries adhere to (at the time) recent GOLD recommendations [20].

In order to identify potentially important patients’ characteristics that distinguish AECOPD patients that require hospitalization compared to those that do not, we conducted a post hoc study from the data collected from the above-mentioned discharge letter analysis [20].

## 2. Materials and Methods

### 2.1. Study Design and Study Participants

We performed a post hoc analysis of the previously conducted retrospective, observational study [20] that assessed COPD exacerbation patients’ discharge records from the emergency department (ED) or following hospitalization.

The primary aim of the above-mentioned study was to evaluate the adherence to GOLD recommendations [20]. Our post hoc analysis was performed in order to focus on the patients’ characteristics to discern premorbid factors and potentially clinically significant elements that might distinguish between AECOPD patients requiring hospitalization and those that could be directly discharged from the ED.

Discharge records of consecutive patients with AECOPD from 8 centers (5 major academic teaching hospitals and 3 county hospitals) in the period from October 2019 to February 2020 were evaluated for the purposes of the initially published research [20]. This study adhered to the Declaration of Helsinki and subsequent amendments and was approved by Institutional Ethics Committee of the University Hospital Dubrava (approval no. 2025/0327-14). The original study was approved by the following Institutional Ethics Committees: University Hospital Centre Zagreb; University Hospital Dubrava; University Hospital Centre Osijek; Clinical Hospital Centre Rijeka, Rijeka; University Hospital Centre Split; Special Hospital for Pulmonary Diseases; Karlovac General Hospital; and Dubrovnik General Hospital. Informed consent was waived by the above-mentioned institutional ethics committee due to the fact that this was a post hoc analysis of previously collected anonymized data from electronic medical records (for which informed consent was also waived [20]).

All discharge letters were evaluated by experienced board-certified pulmonologists by filling out specially designed questionnaires for the purposes of previously published research, as previously described [20].

### 2.2. Data Analysis

Statistical analysis was performed using JAMOVI 1.6.3 and MedCalc Statistical Software version 22.021 (MedCalc Software Ltd., Ostend, Belgium; https://www.medcalc.org; 2024), accessed on 4 January 2025 [21,22].

Categorical variables are presented as numbers and proportions (percentages, %). Parametrically distributed continuous variables are presented as means ± standard deviation (±SD). Nonparametrically distributed variables are presented as medians (first quartile–third quartile) (Q1–Q3).

Comparison between categorical variables between hospitalized patients and those discharged from the ED was conducted using a chi-square test. Formal assessments of continuous variables were conducted using Student’s *t* tests for parametric and Kruskal–Wallis tests for nonparametric variables. Type one error (alpha) was set at 0.05.

Logistical regression modelling was performed in the following manner. An initial (primary) model based on the presumed pathophysiological relevancy was created with the following parameters: Hospitalization was the “event parameter” (dependent variable). Independent variables were age, sex, prior cardiovascular disease (CVD), prior osteoporosis, prior diabetes mellitus, prior long-term oxygen therapy (LTOT), complaint of dyspnea, complaint of fever, complaint of increased frequency of coughing, active smoking status and absolute eosinophil count at admission.

In order to avoid model overfitting, additional models were then created by manually omitting variables with the lowest Wald statistic (“highest *p* value”) using the backward stepwise approach; the final model with 5 degrees of freedom was then reported in the final manuscript. Due to the fact that there were scarce missing data (<1%), missing data were handled by deletion.

The reported (final) model was created with the following parameters: Hospitalization was the “event parameter” (dependent variable). Independent variables were age, prior CVD, prior LTOT, complaint of dyspnea and absolute eosinophil count at admission. The goodness of fit of the model was examined by employing the Hosmer–Lemeshow test. The final model allowed for the reporting of odds ratios for each selected variable (“covariate”).

## 3. Results

### 3.1. Participants’ Characteristics

**Figure 1:**
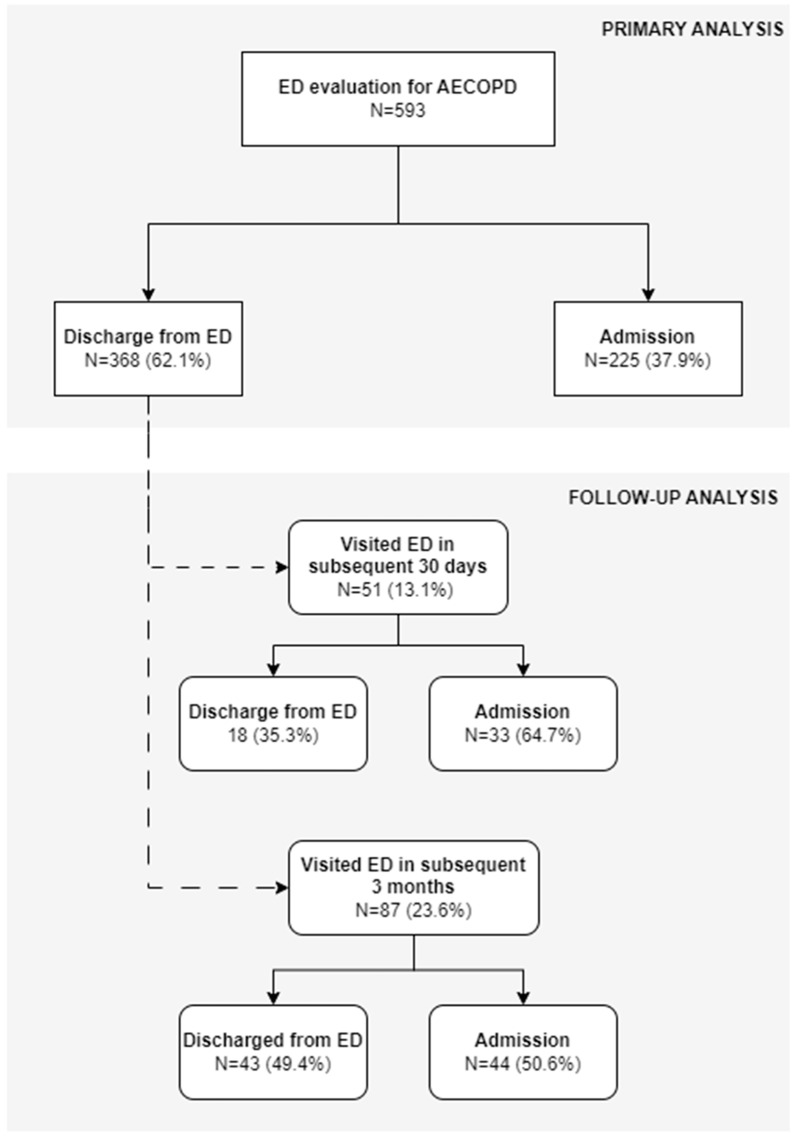
Flowchart depicting main study groups. Primary analysis consisted of patients that underwent emergency department (ED) evaluation for AECOPD, which were then either discharged home from the ED or admitted to the hospital. Follow-up analysis was performed among patients that were discharged (in the primary analysis) and were again evaluated in the ED for AECOPD in subsequent 30 days and in subsequent 3 months from initial ED discharge.

A total of N = 593 COPD patients were included in the analysis. N = 225 (37.9%) were hospitalized, while N = 368 (62.1%) were discharged from the emergency department (ED), as depicted in Figure 1.

Detailed participants’ characteristics are presented in Table 1. Patients had a mean age of 71 (±9.14) years, and 37.4% were female. There were no statistically significant differences between the two groups for age or sex. An increased prevalence of active smoking was observed in the hospitalized group in comparison to the discharged group (38.6% vs. 31.7%, χ^2^ = 1.21, *p* = 0.27). Furthermore, an increased prevalence of prior long-term oxygen therapy (LTOT) was observed in the hospitalized group (19.5% vs. 10.3% for the discharged group, χ^2^ = 9.98, *p* = 0.002).

Most patients had arterial hypertension (N = 384, 64.7%), and almost half (N = 291, 49.1%) had another previously known disease of the cardiovascular system (60.4% of those that were hospitalized vs. 42.1% of discharged patients; χ^2^ = 18.6, *p* < 0.001). Further, 24% of hospitalized patients suffered from diabetes mellitus (vs. 16.8% of discharged patients, χ^2^ = 4.15, *p* = 0.042).

Patients’ symptoms are presented in Table 2. As expected, the vast majority of patients complained of dyspnea (89.5%), one-third complained of fever (33.2% in total; 40% of hospitalized vs. 29.1% of discharged patients, χ^2^ = 8.16, *p* = 0.004) and 76.4% of patients complained of increased cough frequency and/or intensity. Notably, only 28.3% observed a change in the color of their sputum.

Interestingly, patients that were discharged from the ED had a median eosinophil count of 114 (33–230) cells/mm^3^, and those that were hospitalized had lower eosinophils, with a median of 50 (0–187) cells/mm^3^; χ^2^ = 12.8, *p* < 0.001 (Table 2).

### 3.2. Therapy Administered in the Emergency Department

Most patients were treated with SABA (68.3%) and/or SAMA (69.8%), over half received a parenteral systemic corticosteroid (56.6%) and diuretic therapy was administered to about one-fifth of patients (N = 115, 19.4%). In addition, 25.8% of patients that were hospitalized and 15.5% of patients that were discharged received diuretics in the ED (χ^2^ = 3.91, *p* = 0.048). Further, 4.9% of patients received antibiotics in the ED, and 87.1% of hospitalized patients received antibiotic therapy, while 60.1% of discharged patients were prescribed an at-home course of antibiotics (χ^2^ = 53.06, *p* < 0.00001). A summary of the therapy administered in the ED is presented in Table 3.

### 3.3. Logistical Regression Analysis Model

The logistical regression model for hospitalization prediction was statistically significant, χ^2^ = 37.236, *p* < 0.0001. The model was able to correctly classify 68.6% of cases, with an AUC of the ROC curve of 0.703 (95% CI: 0.646–0.756).

Each variable with the pertaining odds ratio and statistical significance is presented in Table 4. When analyzing each variable, eosinophil count and prior LTOT therapy were just outside of the preset margin of statistical significance.

Prior cardiovascular disease was a statistically significant variable (*p* = 0.014): the presence of prior cardiovascular disease increased the odds of hospitalization for 94% (OR 1.94 (95% CI: 1.144–3.3)) in our model.

A subjective complaint of dyspnea was also statistically significant (*p* = 0.005) and increased the odds of hospitalization by 485% (OR 5.85 (1.70–20.14). Conversely, the absence of dyspnea decreased the odds of hospitalization for 83% (OR 0.17 (95% CI: 0.05–0.59)) based on the model we designed.

Age in years was also significant (*p* = 0.033); an increase in age for one year increased the odds of hospitalization for 3% (OR 1.03 (95% CI: 1.003–1.07)) in our model.

### 3.4. Follow-Up of Discharged Patients

Out of 368 patients that were discharged from the ED, N = 51 (13.9%) visited the ED due to AECOPD in the subsequent 30 days, of which 33 (64.7%) required hospitalization (Figure 1). When analyzed for a three-month period, N = 87 (23.6%) of discharged patients visited the ED due to AECOPD in the subsequent 90 days after initial discharge, of which 44 (50.6%) required hospitalization (Figure 1).

At least one ED visit in the subsequent year due to AECOPD was recorded for 134 (36.4%) patients. The median number of AECOPD visits to the ED throughout one year of follow-up among those who had subsequent AECOPD was 1 (1–2).

Interestingly, those that had at least one AECOPD in the subsequent three months had a significantly lower eosinophil count (median 91 (13–160) cells/mm^3^) than those that did not (median 125 (48–257) cells/mm^3^), χ^2^ = 5.92, *p* = 0.015.

## 4. Discussion

We conducted a post hoc study of a retrospective analysis of discharge letters in patients with AECOPD, aiming to identify potentially important characteristics that distinguish patients requiring hospitalization from those that do not.

We did not find statistically significant differences in gender or age between the hospital admission and discharge groups. In a large observational study of hospital readmissions for AECOPD, Chen et al. found that women and men had similar readmission rates for patients younger than 70 years [23]. Conversely, DeMeo et al. demonstrated that younger women were at a greater risk for exacerbations and being categorized as GOLD “D” (according to GOLD 2011 recommendations) [24].

We found a higher prevalence of prior LTOT in hospitalized patients (19.5% vs. 10.3%, *p* = 0.002). Several studies found that LTOT was associated with higher rates of hospitalization and/or readmission [25,26]. No statistically significant differences in the prevalence of arterial hypertension and osteoporosis were found between the two groups. Patients that were hospitalized had a significantly higher rate of prior CVD (excluding arterial hypertension) when compared to the discharged group (60.4% vs. 42.1%, respectively; *p* < 0.001). It has been recognized that the risk of subsequent cardiovascular events and lethal outcomes is significantly increased following AECOPD, especially in frequent exacerbators [15,27,28,29,30].

In terms of symptoms, more patients that required hospitalization complained of dyspnea (93.7% vs. 87%, *p* = 0.001) and fever (40% vs. 29.1%, *p* = 0.004) than patients that were discharged. Patients that were hospitalized had significantly lower eosinophil count levels in the peripheral blood (50 (0–187) vs. 114 (33–230) cells/mm^3^, respectively; *p* < 0.001). Namely, eosinopenia has been recognized to be a poor prognostic factor in the setting of several respiratory illnesses, including community-acquired pneumonia and COVID-19 [31,32,33,34]. In the setting of AECOPD, eosinopenia has also been recognized as an indicator of poor outcomes [35,36]. Steer et al. devised a scoring system (DECAF score), which predicts AECOPD in-hospital mortality, in which eosinopenia (<50 cells/mm^3^) has been recognized as a contributing factor [37,38,39].

Roughly two-thirds of patients received short-acting inhalational therapy in the ED (SABA and/or SAMA), and 56.6% of patients received systemic corticosteroids (46.2% of admitted and 63% of discharged patients, *p* < 0.001). The current commonly used COPD management recommendations such as GOLD do not clearly distinguish between COPD exacerbation phenotypes and do not propose recommendations by which to modify patient management based on a particular underlying exacerbation etiology [40]. A blood eosinophil-guided oral corticosteroid approach takes into account blood eosinophil count. Ramakrishnan et al. conducted a randomized controlled trial, in which they found that the blood eosinophil-guided oral corticosteroid approach in treating AECOPDs is non-inferior to standard practice and may be used in order to reduce systemic glucocorticoid use in clinical practice [41].

About one-fifth (19.4%) of patients received diuretics (25.8% of admitted and 15.5% of hospitalized patients, *p* = 0.048). Namely, underlying heart failure has been recognized as a trigger of AECOPD and is considered to be a distinct AECOPD phenotype by some authors [14,42]. It is, also, clear that heart failure and AECOPDs go “hand in hand” and that one is able to aggravate the other [43,44,45]. Additionally, it has been recognized that it may be difficult to distinguish between heart failure and AECOPD (without heart failure) in COPD patients, especially in the emergency department [46].

Almost all hospitalized patients (87.1%) received antibiotics during hospital stay, and 60.1% of discharged patients were prescribed antibiotics (*p* < 0.00001). Even though antibiotic use without signs of underlying infection remains controversial, a meta-analysis conducted by Suzuki et al., which analyzed randomized controlled trials of antibiotics vs. placebo in the setting of AECOPD, found that antibiotic use was superior to placebo when assessing the frequency of treatment failure. However, they did not find a significant difference in mortality [40,47].

Our post hoc analysis also included a logistical regression model in order to observe factors that contribute towards patient admission. Based on our model, we found that prior CVD, complaints of dyspnea in the ED and age are statistically significant contributors for patient admission. Prior LTOT and eosinophil count (higher eosinophil counts might have a protective role in the model) were just above the pre-set statistical margin. An unadjusted comparison for age did not yield statistical significance. However, our model has indicated age as a statistically significant covariate.

Out of the patients that were discharged from the ED, 13.9% visited the ED due to AECOPD in the subsequent 30 days, of which the majority (64.7%) were admitted. When observing a three-month period, 23.6% visited the ED due to AECOPD, and about half of patients required admission. We found that patients with another ED visit due to AECOPD in a three-month period had significantly lower eosinophil counts (during initial evaluation) than those that did not have additional AECOPDs. This finding is in accordance with the findings of Uslu et al., who also found decreased eosinophil counts in frequent exacerbators [17].

This study was designed as a post hoc study of a retrospective research project that aimed to observe whether physicians complied with the GOLD recommendations and, therefore, has several sources of inherent bias and limitations. Firstly, the primary aim of the study was not to observe patient characteristics, so these data might be imperfect, especially when considering that the source of data is patient histories taken during the ED visit, which are oftentimes less detailed than more detailed histories taken on hospital wards. A second major study limitation is the lack of systemic follow-up, which also highlights the fact that mortality was not investigated and that the patients that did not have subsequent visit(s) to the ED due to AECOPD were lost to follow-up. In addition, data regarding potential subsequent cardiovascular events were not available. Additionally, this study was conducted just before the onset of the COVID-19 pandemic. It is likely that, due to re-organization of the hospital system in Croatia during the pandemic, some patients reported to another ED due to AECOPD. Another limitation is the fact that data regarding COPD severity, presence of emphysema and lung function during stable periods were not available. Additionally, it was not possible to assess COPD severity or distinguish it between hospital admission and ED discharge. Data regarding patients with respiratory failure, respiratory acidosis and management strategies are also lacking.

In spite of the above-mentioned study limitations, our real-world multicentric study provides useful insights into the characteristics of Croatian AECOPD patients, overviews management strategies and identifies eosinophils as potentially protective markers associated with a lower rate of hospital admission. Furthermore, the results of this multicentric collaborative project provide important regional epidemiological information on COPD patients. Future additional studies could explore potential subregional differences among patient populations between different hospital centers.

## Figures and Tables

**Table 1:** Descriptive characteristics of patients with AECOPD with selected comorbidities.

	Total	Discharged from ED	Hospitalized	Statistic	*p*-Value
N	593	368 (62.1%)	225 (37.9%)		
Female sex	222 (37.4%)	139 (37.7%)	83 (36.8%)	χ^2^ = 0.046	0.83
Age (years ± SD)	71 (±9.14)	70 (±8.9)	71 (±9.29)	T = −0.78	0.44
Active smoker	204 (34%)	117 (31.7%)	87 (38.6%)	χ^2^ = 1.21	0.27
LTOT therapy	82 (13.8%)	38 (10.3%)	44 (19.5%)	χ^2^ = 9.98	0.002
Comorbidities					
Cardiovascular disease	291 (49.1%)	155 (42.1%)	136 (60.4%)	χ^2^ = 18.6	<0.001
Osteoporosis	28 (4.7%)	14 (3.8%)	14 (6.2%)	χ^2^ = 1.68	0.2
Arterial hypertension	384 (64.7%)	228 (62%)	156 (69.35)	χ^2^ = 2.78	0.1
Diabetes mellitus	116 (19.6%)	62 (16.8%)	54 (24%)	χ^2^ = 4.15	0.042

ED—emergency department, LTOT—long-term oxygen therapy.

**Table 2:** Patients’ symptoms and eosinophil counts at the time of evaluation in the emergency department.

	Total	Discharged from ED	Hospitalized	Statistic	*p*-Value
Dyspnea	531 (89.5%)	320 (87%)	211 (93.7%)	χ^2^ = 10.3	0.001
Fever	197 (33.2%)	107 (29.1%)	90 (40%)	χ^2^ = 8.16	0.004
Increase in cough	453 (76.4%)	292 (79.3%)	161 (71.5%)	χ^2^ = 3.76	0.053
Productive cough	318 (53.6%)	191 (52%)	127 (54.6%)	χ^2^ = 1.53	0.21
Change in sputum color	168 (28.3%)	100 (27.2%)	68 (30.2%)	χ^2^ = 0.81	0.37
Eosinophilia median (Q1–Q3)	95 (16.8–206)	114 (33–230)	50 (0–187)	* χ^2^ = 12.8	<0.001

* Analyzed with Kruskal–Wallis test; ED—emergency department.

**Table 3:** Therapy administered during the initial evaluation and time observation in the emergency department.

	Total	Discharged from ED	Hospitalized	Statistic	*p*-Value
Antibiotic	419 (70.7%)	223 (60.1%)	196 (87.1%)	χ^2^ = 53.06	<0.00001
Systemic corticosteroid	336 (56.6%)	232 (63%)	104 (46.2%)	χ^2^ = 16.1	<0.001
SABA	405 (68.3%)	262 (71.2%)	143 (63.6%)	χ^2^ = 3.76	0.052
SAMA	414 (69.8%)	268 (72.8%)	146 (64.9%)	χ^2^ = 4.17	0.04
Diuretic	115 (19.4%)	57 (15.5%)	58 (25.8%)	χ^2^ = 3.91	0.048

ED—emergency department, SABA—short-acting beta-2 agonist, SAMA—short-acting muscarinic antagonist.

**Table 4:** Coefficients and standard errors derived from the designed logistical regression model, along with odds ratio and 95% confidence intervals.

Variable	Coefficient ± SE	Wald	*p*-Value	Odds Ratio (95% CI)
Prior LTOT	0.72 ± 0.38	3.62	0.057	2.0454 (0.98–4.28)
Eosinophil count (cells/mm^3^)	−0.002 ± 0.8 × 10^−4^	3.71	0.054	0.998 (0.997–1.0000)
Prior CVD	0.66 ± 0.27	6.04	0.014	1.94 (1.144–3.3)
Complaint of dyspnea	1.77 ± 0.63	7.86	0.005	5.85 (1.70–20.14)
Age (years)	0.03 ± 0.015	4.54	0.033	1.03 (1.003–1.07)
Constant	−4.78 ± 1.29	13.69	0.0002	

CI—confidence interval, CVD—cardiovascular disease, excluding arterial hypertension; LTOT—long-term oxygen therapy.

## Data Availability

The raw data supporting the conclusions of this article will be made available by the authors on request.
